# Profiling Players Involved in Overkill: An Analysis of 71 Cases in Central Italy

**DOI:** 10.3390/healthcare10101873

**Published:** 2022-09-26

**Authors:** Alessandro Mauro Tavone, Giulia Ceccobelli, Giorgia Piizzi, Raimondo Vella, Gabriele Giuga, Andrea Cammarano, Giulia Petroni, Gian Luca Marella

**Affiliations:** 1 Section of Legal Medicine, Department of Biomedicine and Prevention, University of Rome Tor Vergata, 00133 Rome, Italy; 2 Forensic Pathology Section, Department of Surgical Sciences, University of Rome Tor Vergata, 00133 Rome, Italy

**Keywords:** forensic pathology, forensic anthropology, overkill, homicide, victim/offender relationship, violence, offender profiling, victim profiling, injury quantification

## Abstract

“Overkill” is characterized by the finding of excessive wounds on the victim’s body. Despite the large use of this term, it does not have a definition in the literature yet. Our study aimed to analyze the information related to the dynamics of overkill cases, collecting objective variables, and producing a profile of the players involved in this type of homicides. Data on 71 overkill cases from reports of the autopsies performed in the Section of Legal Medicine of the University of Rome Tor Vergata from 1 January 2000 to 31 December 2020 were collected. The victims and the perpetrators of overkill shared similar characteristics: they were usually male aged between 20 and 50 years, more likely in the 20–35 years range; victims however also showed another age peak in the range 50–55 years. The type of damage can be linked to the sole action of a cold weapon or the simultaneous use of multiple harmful tools with no significant differences. The most common motive proved to be a dispute for futile reasons; however, in the case of a single perpetrator of the crime, the economic and passionate motives are as frequent as the previous one. Implications of the findings and avenues for future research are discussed.

## 1. Introduction

Control of interpersonal violence is one of the fundamental tasks of the society. Homicide represents the most serious consequence of violence [1]. The social interest—sometimes more important than the legal one—lies in the fact that homicide represents the destruction of a greater good.

Homicide can operate on the emotional sphere of people, and for this reason, it has constantly stimulated the search for the prominent traits of criminal personality and for the motivations behind it, focusing each time on the social conditions and the prior events, pathological and non-pathological. These research produced a series of theories on violent and criminal behavior, which highlighted how often homicides arose from hidden and unconscious motivations, even if, traditionally, the reasons behind them were included in the classes of rational homicides, explainable on the basis of profit, small social condition (political reasons), and as emotional homicides interpretable on the basis of jealousy, resentment, revenge, or the defense of one’s own honor—the so-called “crimes of passion” [2].

For this reason, the suppression of interpersonal violence cannot ignore the understanding of the underlying structures that generate violence, and therefore keeping track of the epidemiology of violence is a necessity [1].

A particular type of homicide is represented by the so-called “overkill”, which is characterized by the finding of excessive wounds on the victim’s body. Although the term is often used in the literature, it does not have a specific definition. The one, which most authors refer to, is the definition given by Ressler, Douglas and Burgess’s [3], according to which overkill consists of the infliction of more injury than necessary to kill a person. 

Jordan et al. [4] defined overkill as the infliction of multiple wounds to one body area or multiple wounds across many body areas, allowing researchers to code injury severity based on the brutality of the attacker rather than the extent of damage to the victim. 

Tamsen Logan and Thiblin [5], on the other hand, believed that overkill could be differently identified depending on the number and location of the wounds, in relation to the type of injury: 1. A total of 40 or more skin injuries (blunt, sharp, gunshot); 2. three or more sharp wounds located at the head, neck, or trunk with internal organ injuries (including the pleura and large blood vessels); 3. three or more gunshot wounds located at the head, neck, or trunk with internal organ injuries (including the pleura and large blood vessels).

Despite the absence of an exact definition of overkill, attempts to assess injury in general and particularly in cases of overkill, have been made over the years. 

The usefulness of a quantitative analysis on homicide injuries is the possibility to summarize a group of offenders’ behaviors—e.g., sexual offenders—[6] thus facilitating police investigations. They may also be useful in illustrating the nature of a violent crime and the dynamics of the interaction between the offender and victim. This dynamic is known in the law enforcement community where, anecdotally, the level of injury and the location of those injuries on the body have implied some level of existing relationship [7,8,9,10].

The appropriate classification of injuries by type and severity is fundamental to the study of injury etiology. Scales for categorizing injuries are grouped into two types: those that assess the physiological status and those that describe the injury in terms of anatomical location, specific lesion, and relative severity [11,12,13].

The most important classification for our study is the “Homicide Injury Scale” (HIS), that was developed to capture the overkill features or excessive injuries on the victims [6]: overkill features, for this purpose based on the multiple examples given by Douglus et al. [14] are classified as an HIS score of 5 or 6, as shown in Table 1 [6].

HIS is an alternative measure to the classical scores (such as ISS–Injury Severity Score and NISS–New Injury Severity Score), proffered for the purpose of providing a quantitative standard measure of the degree of injury. 

Delineating the nature of the injuries may prove more useful in differentiating offender variables. The need for differentiation and, therefore, more specificity and accuracy in victim injury analysis is what led to the development of a scheme for measuring homicidal injuries—the HIS. 

This measure can be used to compare injuries across a large sample of cases for which different and multiple causes of death and attendant levels of injury exist. The HIS attempts to further capture the qualitative element of a victim’s fatal injuries (within the context of homicide) in a quantitative manner. This is accomplished by quantifying the severity of only those injuries that are directly related to the cause(s) of death through a detailed review of the medical examiner’s autopsy report and related protocols. Following this scheme, the HIS was formulated to capture the number of causes of death and related injury severity as identified by a medical examiner. The proposed values and attendant interpretations for the HIS are shown in the overmentioned Table 1 [6].

Overkill is an example of forensic finding that will often lead the investigator to a specific homicide category and, thus, a possible motive for the offense [14]. Hence, the medical examiner and investigators have an essential role in evaluating not only the number of injuries, but also their distribution, morphology, and the weapon used to inflict them [15]. 

To obtain a classification in the single specific case, it is mandatory to univocally identify the presence or absence of the overkill. 

According to Trojan, Salfati, and Schanz [16], the lack of a concrete and objective definition of overkill impedes proper operationalization of the term and complicates replication across studies and the direct comparison of results, therefore limiting our ability to empirically determine its full utility and application.

In addition to the classic, more objective and quantifiable characteristics within a crime scene—e.g., number and locations of wounds, simultaneous use of multiple means—many variables are reported in the literature to identify overkill.

For example, it is not only the number of wounds that defines it, but also their severity. 

Even cases with a single wound were somehow classified as overkill, whereas a nearly complete penetration of the weapon—including the handle—into the body of the victim was recorded [17].

According to some other authors, clear examples of overkill could be the following: a victim so brutally beaten with a blunt object to leave impressions all over the head; a neonate with blunt lesions on the cranium associated with several stabs in the neck and the thorax [18], as well as victims shot twice in the head [19,20].

Fritzon and Ridgway [21] suggested that victim’s level of resistance to an attack may contribute to the level of violence in a homicide, and therefore the action of the victim should be considered in suspect of an overkill.

Another critical issue that has been raised is whether the definition of overkill should consider that if the murderer is aware of committing an extremely violent act, that is, inflicting on the victim more wounds than necessary to kill, or if the perpetrator of such a crime is driven by a strong emotional state, such as anger or loss of control. In fact, if awareness becomes a necessary requirement in the definition, this can prove problematic when the murderer acts in the grip of an intensified emotional state and is therefore not aware of inflicting an excessive number of wounds on the victim [16].

In this regard, Beauregard and Martineau [22] sustained that some organized murders perpetrated overkill to prevent the identification of the victim’s identity.

According to Keppel et al. [23], probably the perpetrator who overkills is aimed to have complete control and domination over the victim. Interestingly, the author also associated picquerism with the overkill, as a paraphilia and a form of sadism consisting in the sexual interest in penetrating the skin of another person sometimes enough to cause the death.

The importance of the relationship between victim and offender seems debated. Douglas et al. [24] stated “the more evidence there is of overkill, the closer the relationship is between the victim and the offender”, suggesting to investigators that if overkill was present, they should have concentrated their investigation on those known to the victim [16] while empirical studies [10,25] demonstrated that excessive injuries were not related to offender–victim relational categories. 

For many authors, the use of variable with clear, objective, and observable definitions should be preferred to the descriptions of subjective ones and focused on the internal motivation of the offenders, that may be open to personal interpretation [26,27,28].

This led to different trends in the empirical homicide literature in which overkill was examined as a distinct variable of crime scene investigation versus the use of simpler, but more objective concepts, such as the number of inflicted wounds, adding more difficulties in operationalizing the term.

Our study aims to analyze the information relating to the dynamics of the overkill cases present in the records of the Section of Legal Medicine of the University of Rome Tor Vergata, collecting objective variables and producing a profile of the characteristics of the victims and perpetrators most involved in this type of homicides.

## 2. Materials and Methods

A retrospective analysis of all autopsy reports performed in the Section of Legal Medicine of the University of Rome Tor Vergata from 1 January 2000 to 31 December 2020 was conducted. After a first screening, all non-homicide reports were discarded. For single case eligibility, the following inclusion criterion was adopted: reports about cases of overkill, defined by at least one of the three definitions given above [3,4,5], as suggested by Jordan. 

During the study period, a total of 153 homicide cases were autopsied and only 71 cases fitting our criterion were selected.

A data extraction sheet was developed and for each single case the following items were recorded: details of the victims and autoptic findings (age; sex; number, kind, and location of the wounds; site where the body was found); details of the perpetrator (age; sex; relationship with the victim; motive; psychiatric pathologies; wounding means used).

It is important to note that for cases in which one victim had more than one perpetrator, or perpetrator remained unknown, or one perpetrator killed more than one victim were properly recorded, and a single extraction sheet was made for each individual (victims and perpetrators).

For each feature, a descriptive analysis was performed using software SPSS 26. Data were also divided considering if the murders were committed by one, or more, or unknown perpetrator. Finally, we analyzed the cases by considering four categories: 1. All cases 2. Single perpetrator 3. Multi perpetrator 4. Perpetrator unknown. For the qualitative variables, absolute percentages and frequencies were calculated. Quantitative variables were investigated by the mean values and the standard deviation.

## 3. Results

**Figure 1:**
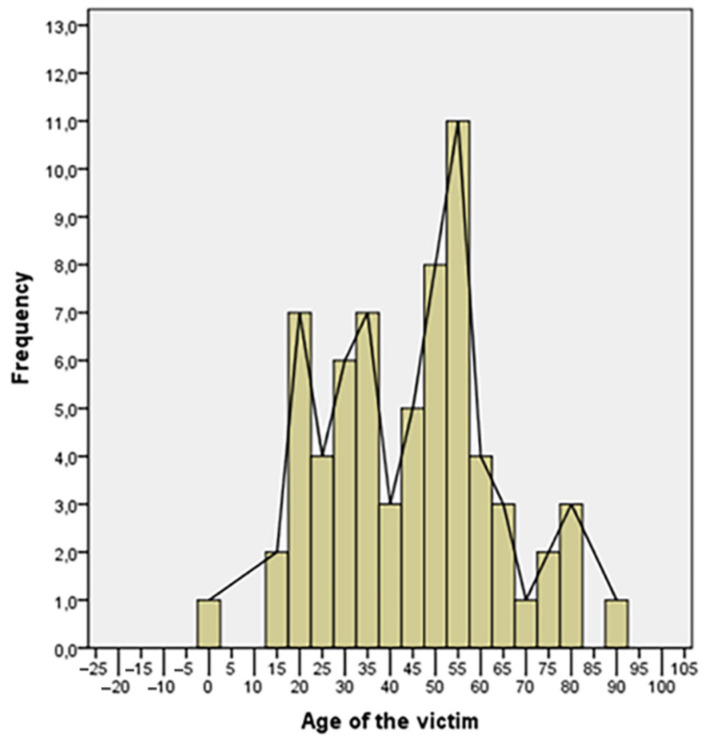
Age distribution of the victims.

**Figure 2:**
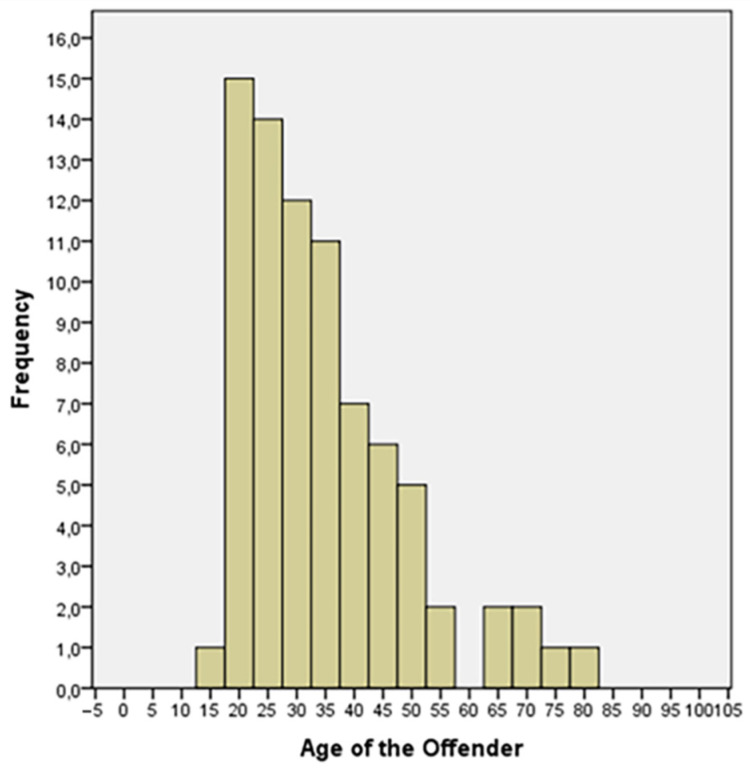
Age distribution of the offenders.

The results of statistical analysis are summarized in the following tables (Table 2 and Table 3) and graphically shown in Figure 1 and Figure 2.

### 3.1. Victims

Among the 71 cases that were analyzed, the total number of victims was 73, because of the presence of 2 cases of double homicide. In addition, 41 cases presented only one murderer. 

### 3.2. Perpetrators

In 18 cases there was more than one perpetrator of the crime, for a total of 52 perpetrators (six homicides committed by two perpetrators, eight homicides committed by three perpetrators, and four homicides committed by four perpetrators). In 12 cases, no information on the perpetrator was available.

The total number of perpetrators identified was therefore 93 in a total of 59 cases. For 3 of the 93 identified perpetrators, all of whom involved in the same homicide, it was impossible to find information about sex and age.

## 4. Discussion

The analysis of the data deriving from the forensic work, through a careful examination of the victim’s body and the crime scene, integrated with circumstantial data of the methodologies and dynamics of the killing, are considered fundamental in the definition of criminal profiles and personality characteristics of the offender in order to direct police investigations [24,29,30].

Although overkill has aroused the interest of many authors in the last 20 years, its investigation cannot be considered exhausted yet, as proved by the heterogeneity of the definitions suggested over the years. 

Being an extremely complex and diversified entity, the approach to overkill cannot leave out of consideration the record of all the characteristics of the actors, which are not only limited to the harmful patterns and the tools used, but includes the analysis of the relationships, motivations, contexts, and all the circumstantial data.

A type of killing characterized by extreme violence, such as overkill, tends to manifest itself in specific geo-political and socio-cultural contexts.

For some authors, homosexuals, but also subjects belonging to ethnic minorities, constitute a category of subjects particularly at risk for an overkill [31,32]; this is especially true for subjects who most frequently indulge in casual relationships and with strangers [33,34,35]. According to Bell and Vila [36], from the comparison between homicides among homosexuals and heterosexuals, a clear prevalence of overkill was noted in the formers. 

Among our cases, there were four homosexual male victims.

According to Zara et al. [37], another category at risk for violent murder is represented by prostitutes, especially when the connection with the murderer was not limited to the sole relationship of customer/service provider but was of a more intimate nature. In our records, as well, there were three cases with prostitutes among the victims. In two of these cases, the motive was of a crime of passion: the killers were respectively the victim’s boyfriend and a regular customer. In the third case, the motive was of an economic nature, as the regular customer refused to pay the woman.

Other particular socio-cultural contexts are those highlighted by Toprak and Ersoy [38], who argued that one in three femicides in Turkey had the characteristics of an overkill. Adinkrah [39] described the use of excessive violence in the murders perpetrated by young Ghanaian men against their grandmothers, whom they accused of witchcraft and of having been responsible for their problems, to be mostly the result of an economic and/or physical motive, with the goal of annihilating the “witch” and recovering the lost well-being.

It should also be noted that 21 (28.8%) victims and 35 perpetrators among those identified (37.6%) in our cases were immigrants (generally from Eastern Europe or South-Asia).

As highlighted by Potenza et al. [40] about feminicide—including, though a broader definition than the WHO’s one, any killing of women or girls -: “*contrary to what may seem from the frequency and animosity of public opinion debates and from the media, which report a steady increase in the number of women murders, these events do not seem to be an emerging phenomenon of recent years but, in accordance with national statistical data, they remained stable*”.

In our cases, only 18 out of 73 (24,66%) victims were women, indicating that the increase of violence in overkill was not related to gender violence and feminicide.

An emerging problem comes from the world of online relationships, where victims are killed during their first meeting, by a person met via social networks. These killings were usually characterized by a high number of wounds mainly caused by cold weapons, distributed on various parts of the body, many of whom on the chest and, sometimes, by mutilation [41]. In our records, this was the case for one of the murders of a homosexual, in which the victim and the killer had met through social media, and the homicide was committed at the end of their first date, when the murderer tried to rob the victim.

Another important relationship to be considered is the one between overkill and the psychiatric pathologies of the killers. A study by Laajasalo and Hakkanen [42] analyzed 125 murders committed by subjects suffering from schizophrenia: in 37 cases (29.6%) there was excessive use of violence, especially in cases that occurred in the family, as also confirmed by Kondo and Oshima [43]. In the latter type of homicides, the level of violence was extreme, as in the case of a son with schizophrenia who killed his mother with 85 stabs on the face, limbs, chest, and abdomen, and finally decapitated her [44]. 

Among our cases, five offenders (5.4%) were affected by a psychiatric pathology, four of whom by a psychotic disorder with delusions and hallucinations, and one with an attention disorder with hyperactivity associated with a learning disorder. Although psychotic disorders seem to be the most frequent ones, it is also possible to find associations between overkill and personality disorders (borderline, antisocial, or schizoid or paranoid traits) [45], in whom the behavior of the perpetrator is in their mind justified by the interpretation they have of the relationship with others, who are turned into inanimate entities, whose sacrifice is justified by the achievement of the interests of the killer, in the absence of guilt [2].

Finally, a case of homicide-suicide present in our records opened to interesting psychodynamic considerations: a woman, a medical oncologist, received continuous proposals and invitations from the son of one of her patients, who had had fantasies about the possibility of a romantic relationship between them, misunderstanding the availability derived from her working role. After numerous refusals, the man waited for the doctor in the hospital parking lot and then killed her by stabbing her 25 times. Once back home, he took his own life by strangling himself with two electrical cable wires. In this case, two different distinctive traits emerged. On the one hand, a lack in controlling the aggressive, irrepressible impulse, deriving from anger due to rejection, to which other people would have responded in a more adequate and understandable way. This characteristic, according to Andransen and Black [46], is notoriously associated with violent behaviors. On the other hand, given the brutality, but above all the self-incrimination expressed in this case by the suicide, are the distinctive traits of the depressed man [47]. 

This theory, however, deviates from the classic interpretation made by Dalmas (cited by Franchini) [48], in which the depressed person, who is trapped in a narrow, hostile “space of life”, with no way out and no future prospects, decides to take his own life and to take it away from the dearest person, who is, in the mind of the perpetrator, also caged in an unsustainable existential condition.

Concerning the wounds and the weapons used, it should be noted that in many cases (32.9%) more than one harmful tool was used, almost always represented by the combination of cold weapons and blunt tools. 

However, the use of cold weapons appeared predominant, especially if compared with that of firearms. These data were not surprising given that the killings of the research sample occurred in Italy, where the possession of firearms is limited by law, and there are only six cases in the literature [2,45]. 

This trend is also confirmed by the international literature, as we can only find one article that reports two murders, occurred in Canada and perpetrated with the use of firearms, committed by the same killer and with the same weapon, which were categorized by the authors as overkill [21]. Lastly, in eight cases the perpetrators set the body on fire after committing the overkill. This similar circumstance appears to be uncommon in the literature as it is only found in another case [2]. 

It is also important to consider the use of the so-called “weapons of opportunity” [49], i.e., a commonly used tool that can be used as a weapon, if necessary. In our records, as well, there were cases with these characteristics: tools, always used in combination with others, such as sticks, iron bars, and in one case, a plastic tube used to strangle the victim. This case, which according to our previous work was the most common among the overkill cases (14 cases out of 25), would seem to play an important role in transforming an aggressive impulse, which would otherwise end in a less serious criminal offense, into a violent homicide, favoring the interpretation that these homicides represent an involuntary outcome, or at least not premeditated, resulting from an impulsive and uncontrollable reaction, which in the absence of favorable circumstances, would not have materialized [45].

## 5. Conclusions

Based on the frequencies that emerged from our analysis, it is possible to reconstruct a profile of the most common characteristics of the victims, perpetrators, and their modus operandi.

### 5.1. Profile of the Victim 

The victim of overkill cases was usually a male adult aged between 20 and 35 years, or between 50 and 55 years (64% of cases fall into one of these age groups). The number of wounds on the body was usually remarkable, an average of 18, and they are typically localized in the chest and upper limbs, probably as a result of defense. 

The type of damage could be linked to the sole action of a cold weapon or the simultaneous use of multiple harmful tools with no significant differences. With a similar percentage, the victim’s body was usually found inside their home or on the street.

### 5.2. Profile of the Perpetrator

In more than 90% of the cases, the perpetrator of an overkill was an adult male aged between 20 and 50, more likely in the 20–35 range (65% of cases). If more than one person was involved in the murder, they were usually acquaintances/friends of the victim. When the crime was committed by a single person, there was a significant increase in family or sentimental relationships between the killer and the victim. When the perpetrator and the victim were strangers, the action was usually committed in a group, and it was linked to mafia or criminal clans’ activities. The most common motive proved to be a dispute for futile reasons; however, in the case of a single perpetrator of the crime, the economic and passionate motives were as frequent as the previous one.

## Figures and Tables

**Table 1:** The Homicide Injury Scale (HIS) according to Safarik et al., 2005 [6].

The Homicide Injury Scale (HIS)	
1	Single cause of death only: internal injuries only with no visible related external injuries (e.g., smothering, strangulation, ruptured organs resulting from blunt force trauma)
2	Single cause of death only: internal injuries only with minor related external injuries (e.g., smothered with related abrasions and/or contusions of mouth and face, strangled with related abrasions or ligature marks)
3	Single cause of death only: related external moderate to serious injuries not identified as either excessive or overkill
4	Two or more causes of death: related internal and/or external injuries not identified as either excessive or overkill
5	Single cause of death only: related external injuries identified as either excessive or overkill
6	Two or more causes of death: related internal and/or external injuries in at least one of the causes of death identified as either excessive or overkill

**Table 2:** Descriptive statistics of the victims.

VICTIMS	All Cases (N = 73)	Single Perpetrator (N = 43)	Multi- Perpetrator (N = 18)	Perpetrator Unidentified (N = 12)
Sex (Female)	18 (24.66%)	16 (37.21%)	0 (0.0)	2 (16.66%)
Age *	44.59	47.70	34.56	47.55
	(SD 18.55)	(SD 18.72)	(SD 11.13)	(SD 20.84)
Total number of wounds (average) *	18.10 (SD 18.82)	18.03 (SD 22.7)	18.73 (SD 11.53)	17.45 (SD 8.95)
Charred body	8 (10.96%)	5 (11.63%)	3 (16.66%)	0 (0.0 %)
Signs of a struggle *	38 (52.05 %)	19 (44.19%)	13 (72.22%)	6 (50%)
Location of the wounds:				
- Head	56 (77.71%)	34 (79.07%)	13 (72.22%)	9 (75%)
- Neck	41 (56.16%)	26 (60.46%)	11 (61.11%)	4 (33.33%)
- Chest	61 (83.56%)	36 (83.72%)	16 (88.88%)	9 (75%)
- Abdomen	31 (42.46%)	18 (41.86%)	9 (50.00%)	4 (33.33%)
- Upper limbs	61 (83.56%)	34 (79.07%)	16 (88.88%)	11 (91.6%)
- Lower limbs	44 (60.27%)	21 (48.84%)	13 (72.22%)	9 (75%)
Site of body dump:				
- Public street	24 (32.87%)	11(25.58%)	8 (44.44%)	5 (41.7%)
- Workplace	7 (9.59%)	6 (13.95%)	1 (5.55%)	0 (0.0%)
- House	26 (34.25%)	18 (41.86%)	4 (22.22%)	4 (33.3%)
- Concealed	15 (20.55%)	8 (18.60%)	5 (27.77%)	2 (16.7%)
- Unknown	1 (1.37%)	0 (0.0%)	0 (0.0%)	1 (8.3%)
Wounding means used:				
- Firearms wounds	13 (17.8%)	8 (18.60%)	1 (5.55%)	4 (33.3%)
- Multiple wounds:	24 (32.87%)	14 (32.56%)	7 (38.88%)	3 (25%)
Blunt + incised	12			
Blunt + asphyxia	6			
Blunt + firearms	3			
Incised + asphyxia	1			
Blunt + incised + asphyxia	1			
Blunt + incised + firearms	1			
- Incised wounds	27 (36.98%)	20 (46.51%)	5 (27.77%)	2 (16.66%)
- Blunt force wounds	9 (12.32%)	1 (2.33%)	5 (27.77%)	3 (25%)

* Including records with missing data.

**Table 3:** Descriptive statistics of the perpetrators.

Perpetrators	All Cases (N = 73)	Single Perpetrator (N = 43)	Multi- Perpetrator (N = 18)	Perpetrator Unidentified (N = 12)
Sex (Male/Female)	84 M / 6 F	38 M / 3 F	46 M / 3 F	-
	(SD 14.2:1)	(SD 12.6:1)	(SD 15.6:1)	
Age *	34.9	39.92	30.0	-
	(SD 14.2)	(SD 16.05)	(SD 9.97)	-
Relationship victim/perpetrator				
- Sentimental	7 (7.5%)	7 (17.07%)	0 (0.0%)	-
- Family unit/ cohabiting	17 (18.3%)	10 (24.4%)	7 (13.46%)	-
- Other relatives	3 (3.2%)	3 (7.32%)	0 (0.0%)	-
- Acquittance/friend	40 (43%)	16 (39.02%)	24 (46.15%)	-
- Strangers:	17 (18.3%)	4 (9.76%)	13 (25%)	-
Mafia	4 (4.3%)	0 (0%)	4 (7.7%)	-
Criminal clans	2 (2.1%)	0 (0%)	2 (3.85%)	-
Occasional customer	1 (1.1%)	1 (2.44%)	0 (0.0%)	-
- Unknown	9 (9.7%)	1 (2.44%)	8 (15.4%)	-
Motive of the crime:				
- Crime of passion	13 (14%)	10 (24.4%)	3 (5.7%)	-
- Futile motives	37 (39.8%)	10 (24.4%)	27 (52%)	-
- Psychiatric pathology	5 (5.4%)	5 (12.2%)	0 (0.0%)	-
- During other crime	15 (16.1%)	3 (7.3%)	12 (23.1%)	-
- Economic	19 (20.4%)	12 (29.3%)	7 (13.5%)	-
- Unknown	4 (4.3%)	1 (2.4%)	3 (5.7%)	-

* Including records with missing data.

## Data Availability

Data available on request due to restrictions e.g., privacy or ethical The data presented in this study are available on request from the corresponding author.
